# Differences in Brain Volume between Metabolically Healthy and Unhealthy Overweight and Obese Children: The Role of Fitness

**DOI:** 10.3390/jcm9041059

**Published:** 2020-04-08

**Authors:** Cristina Cadenas-Sanchez, Irene Esteban-Cornejo, Jairo H. Migueles, Idoia Labayen, Juan Verdejo-Román, Jose Mora-Gonzalez, Pontus Henriksson, José Maldonado, José Gómez-Vida, Charles H. Hillman, Kirk I. Erickson, Arthur F. Kramer, Andrés Catena, Francisco B. Ortega

**Affiliations:** 1 PROFITH “PROmoting FITness and Health through Physical Activity” Research Group, Department of Physical and Sports Education, Faculty of Sport Sciences, University of Granada, 18071 Granada, Spain; ireneesteban@ugr.es (I.E.-C.); jairohm@ugr.es (J.H.M.); jmorag@ugr.es (J.M.-G.); ortegaf@ugr.es (F.B.O.); 2 MOVE-IT Research Group, Department of Physical Education, Faculty of Education Sciences University of Cádiz, 11519 Cádiz, Spain; 3 Institute for Innovation & Sustainable Development in Food Chain (IS-FOOD), Public University of Navarra, 31006 Pamplona, Spain; idoia.labayen@unavarra.es; 4 Center for Cognitive and Brain Health, Department of Psychology, Northeastern University, Boston, MA 02115, USA; c.hillman@northeastern.edu (C.H.H.); a.kramer@northeastern.edu (A.F.K.); 5 Mind, Brain and Behavior Research Center (CIMCYC), University of Granada, 18011 Granada, Spain; j.verdejo@gmail.com; 6 Laboratory of Cognitive and Computational Neuroscience (UCM-UPM), Center for Biomedical Technology (CTB), Pozuelo de Alarcón, 28223 Madrid, Spain; 7 College of Health and Human Services, University of North Carolina at Charlotte, Charlotte, NC 28233, USA; 8 Department of Biosciences and Nutrition at NOVUM, Karolinska Institutet, 14183 Huddinge, Sweden; pontus.henriksson@liu.se; 9 Department of Health, Medicine and Caring Sciences, Linköping University, 58183 Linköping, Sweden; 10 Department of Pediatrics, School of Medicine, University of Granada, 18016 Granada, Spain; jmaldon@ugr.es; 11 The Institute of Biomedicine Research (Instituto de Investigación Biosanitaria (IBS)), 18014 Granada, Spain; 12 Department of Pediatrics, San Cecilio Hospital, 18016 Granada, Spain; gomezvida@gmail.com; 13 Department of Physical Therapy, Movement, & Rehabilitation Sciences, Northeastern University, Boston, MA 02115, USA; 14 Brain Aging & Cognitive Health Lab, Department of Psychology, University of Pittsburgh, Pittsburgh, PA 15260, USA; kiericks@pitt.edu; 15 Beckman Institute, University of Illinois at Urbana-Champaign, Champaign, IL 61801, USA; 16 Department of Experimental Psychology, Mind, Brain and Behavior Research Center (CIMCYC), University of Granada, 18011 Granada, Spain; acatena@ugr.es

**Keywords:** academic achievement, cardiorespiratory fitness, global volume, gray matter, regional volume

## Abstract

The aim of this study was to examine whether metabolically healthy overweight/obese children have greater global and regional gray matter volumes than their metabolically unhealthy peers. We further examined the association between gray matter volume and academic achievement, along with the role of cardiorespiratory fitness in these associations. A total of 97 overweight/obese children (10.0 ± 1.2 years) participated. We classified children as metabolically healthy/unhealthy based on metabolic syndrome cut-offs. Global and regional brain volumes were assessed by magnetic resonance imaging. Academic achievement was assessed using the Woodcock-Muñoz standardized test. Cardiorespiratory fitness was assessed by the 20 m shuttle run test. Metabolically healthy overweight/obese (MHO) children had greater regional gray matter volume compared to those who were metabolically unhealthy (MUO) (all *p* ≤ 0.001). A similar trend was observed for global gray matter volume (*p* = 0.06). Global gray matter volume was positively related to academic achievement (*β* = 0.237, *p* = 0.036). However, all the associations were attenuated or disappeared after adjusting for cardiorespiratory fitness (*p* > 0.05). The findings of the present study support that metabolically healthy overweight/obese children have greater gray matter volume compared to those that are metabolically unhealthy, which is in turn related to better academic achievement. However, cardiorespiratory fitness seems to explain, at least partially, these findings.

## 1. Introduction

Childhood obesity is one of the major health concerns of this century as it has reached epidemic proportions worldwide [1]. These are alarming results since an excess of fat mass has well-known comorbidity with other physical health parameters, including insulin resistance, hypertension, and type-2 diabetes [2,3], as well as mental health problems and measurable changes in brain and academic achievement [4,5,6,7]. A recent meta-analysis highlights that obesity and body mass are related to significantly lower gray matter volume in brain areas underlying executive function [8]. Likewise, Gracia-Marco et al. [9] investigated the associations of obesity with gray and white matter volumes, concluding that only lean mass index was positively associated with white matter volume and to a lower extent with gray matter volume in certain regions of the brain. However, there is a subset of the population, referred to as metabolically healthy overweight/obesity (abbreviated as MHO to refer to both overweight and obese children with a healthy metabolic profile), who do not have these metabolic abnormalities. The MHO phenotype is defined as an excessive body weight based on the international body mass index (BMI) cut-points, but otherwise do not meet any other metabolic syndrome criteria (i.e., triglycerides, glucose, high-density lipoprotein, and systolic and diastolic blood pressure) [2] except for waist circumference. This can be identified in children using the age- and sex-specific cut-points proposed by Jolliffe and Janssen [10].

Recent systematic reviews and meta-analyses have concluded that MHO individuals are at a higher risk of cardiovascular mortality and morbidity than those who are metabolically healthy normal-weight [11,12,13,14,15]. However, it has been shown that variability in cardiorespiratory fitness could at least partially explain these effects [2,16,17]. Within the same weight status, MHO individuals are characterized by having lower amounts of visceral adipose tissue and adipose cell size, as well as higher cardiorespiratory fitness and physical activity levels, than metabolically unhealthy overweight/obesity (MUO) [2,16,18,19,20]. Nevertheless, whether metabolic differences between MHO and MUO may also extend to brain and academic achievement in young individuals remains unexplored.

In addition, given the major role of cardiorespiratory fitness in the characterization and prognosis of the MHO phenotype [2,16,17,21] and the link between cardiorespiratory fitness and gray matter demonstrated in our previous study [22], it is important to consider the role of cardiorespiratory fitness in the association of the MHO phenotype with brain and academic outcomes. Therefore, the aims of this study were: (1) to examine whether MHO individuals had greater global and regional gray matter volumes than their MUO peers; (2) to examine the associations between gray matter volume and academic achievement as a function of metabolic health status; and (3) to examine whether cardiorespiratory fitness modifies these differences and associations among overweight/obese children.

## 2. Material and Methods

### 2.1. Design

This study used baseline data from the ActiveBrains Project (http://profith.ugr.es/activebrains, Clinical Trial identifier: NCT02295072). Briefly, ActiveBrains is a randomized controlled trial designed to examine the effect of a physical activity intervention on brain, cognition, academic achievement, and physical and mental health in overweight/obese children. Baseline data collection occurred between 2014 and 2016. Detailed information about the project protocol has been described elsewhere [23].

### 2.2. Participants

A total of 97 children (10.0 ± 1.2 years old, 60 boys; MHO *n* = 52) with valid baseline data were included in this study. The inclusion criteria were that children had to be 8–11-years-old, right-handed (due to differences in brain measures between left- and right-handed), overweight or obese based on the World Obesity Federation’s cut-offs (formerly named International Obesity Task Force, IOFT) [24,25], without psychological or physical disorders, and, for girls, not to have onset of menarche. We obtained informed consent from parents or legal guardians after the objectives and measurements of the project had been explained by a researcher. The study followed the ethical guidelines of the Declaration of Helsinki 1964 (revised Edinburgh 2000) and was approved by the Ethics Committee on Human Research (CEIH) of the University of Granada (reference number: 848).

### 2.3. Measurements

#### 2.3.1. Anthropometric Variables

We measured the participants’ weight (kg) and height (cm) (SECA, Hamburg, Germany) in underwear and without shoes and calculated the BMI (kg/m^2^). Overweight and obesity type I, II, and III were classified based on the sex- and age-specific BMI cut-points supported by the World Obesity Federation [24,25]. We collected all anthropometric measurements twice, and we recorded the mean value for analyses.

#### 2.3.2. Blood Analyses and Blood Pressure

We measured metabolic risk factors, such as serum triglyceride concentration, glucose, and high-density lipoprotein cholesterol, from fasting blood samples collected at the hospital between 8:00 a.m. and 10:30 a.m. Systolic and diastolic blood pressures were measured in a sitting position from the left arm with an automatic sphygmomanometer (Omron M6, Hoofddorp, The Netherlands). For blood pressure, two measurements were collected on different days, and the minimum value was registered for the analyses.

#### 2.3.3. MHO and MUO’s Categorization

Based on previous reviews conducted by our group [2,16], children were characterized as MHO if they did not present altered values for any of the following four risk factors: triglycerides, glucose, high-density lipoprotein, and systolic and/or diastolic blood pressure. Waist circumference was excluded as a criterion since most of the overweight/obese individuals presented higher waist circumference [16]. On the contrary, children were characterized as MUO if they presented one or more values indicating metabolic abnormalities, i.e., one or more altered values for the risk factors mentioned above. In this study, when we refer to MHO and MUO, we are including both overweight and obese children due to our relatively small sample size (*n* = 97). The supplementary material includes sensitivity analyses conducted only in obese children (*n* = 73).

Cut-offs for metabolically healthy and unhealthy categorization were based on the age- and sex-specific cut-points for an adolescent population (from 12- to 18-years-old) provided by Jolliffe and Janssen [10]. Since the age range of our sample was from 8- to 11-years-old, we used the closest cut-offs provided for boys and girls aged 12 (see Table S1). The strengths of these cut-offs are linked to the International Diabetes Federation and Adult Treatment Panel and adapted for age- and sex-specific metabolic syndrome criteria based on growth curves.

#### 2.3.4. Global and Regional Brain Volume

Brain volume was assessed by magnetic resonance imaging (Siemens Magnetom Tim Trio, 3T, Siemens, Erlangen, Germany). We collected a high resolution sagittal three-dimensional T1-weighted image using magnetization-prepared rapid gradient-echo sequence (MPRAGE) [22]. The total acquisition time for the T1 sequence was 7 min and 31 s for each child.

Structural images were pre-processed using the Statistical Parametric Mapping software (SPM, version 12, Wellcome Department of Cognitive Neurology, London, United Kingdom). Firstly, we checked the T1-images for each participant to detect any artefacts/movement, and we aligned the image to the anterior and posterior commissure. After this first screening, we segmented the T1-weighted structural images into gray matter, white matter, and cerebrospinal fluid [26]. Secondly, we created a template using Diffeomorphic Anatomical Registration Through Exponentiated Lie algebra (DARTEL) [27] based on gray and white matter tissues images. After the template creation, DARTEL estimates the best set of smooth deformations for every child’s tissue to the common average, applies the deformations to create a new average, and finally reiterates the process until convergence. The resultant images were spatially normalized to the Montreal Neurological Institute (MNI) space with affine transformation to create the DARTEL template. Each participant’s segmented images were normalized to the DARTEL template. To perform a volume change correction, we modulated the normalized gray matter images (each voxel) with the Jacobian determinants derived from spatial normalization [28]. Lastly, the volumetric images were smoothed by convolution with an isotropic Gaussian kernel of 8 mm full-width at half-maximum (FWHM). The calculation of the global brain volume was derived by using the non-normalized segmented images (gray and white matter tissue).

#### 2.3.5. Academic Achievement

Academic achievement was measured using the Spanish adaptation of the Woodcock-Johnson III (i.e., Woodcock-Muñoz standardized test), which is a validated measure of academic achievement [29]. This test comprises a standard battery of academic components, such as language and mathematics. For this study, we selected total academic achievement as an overall score. The Woodcock-Muñoz test was administered individually by a trained evaluator. The testing lasted between 100 and 120 min per child. All data were recorded in the Compuscore and profile software version 3.1 (Riverside Publishing Company, Itasca, IL, USA) and extracted in standard scores.

#### 2.3.6. Cardiorespiratory Fitness

Cardiorespiratory fitness was evaluated by a 20 m shuttle run test. In brief, children ran a 20 m distance following an audio signal. The initial speed was 8.5 km/h with 0.5 km/h/min increment per stage. The test concluded when children stopped due to exhaustion or failed to reach the end lines concurrent with the audio signal on two consecutive occasions. Maximum oxygen consumption (VO_2_max) was calculated based on the Leger et al. [30] equation (Y = 31.025 + 3.238X − 3.248A + 0.1536AX, where A corresponds to age and X to the last stage completed).

#### 2.3.7. Other Covariates

Sex, peak height velocity, parental education level, and BMI were included in the models as basic confounding variables. The peak height velocity is one of the most commonly used indicators for biological maturation (i.e., biological age). Peak height velocity was calculated from height or seated height using a sex-specific equations: for boys, −8.13 + (0.007 × (age × seated height)); for girls, −7.71 + (0.004 × (age × height)) [31]. Chronological age replaced peak height velocity as a confounder when academic achievement was included as the main dependent variable in the model, since it had the highest predictive value, while peak height velocity was the strongest for brain outcomes. The parents’ educational level was self-reported. The parents reported their maximum educational level achieved, and the responses were combined as: neither parent had a university degree, one of them had a university degree, and both of them had university degrees.

Based on our most recently published study [22], and considering the role of cardiorespiratory fitness component on health, as well as on a metabolically healthy phenotype [2,18,20,21], we additionally included cardiorespiratory fitness (VO_2_max) as a confounder in fully adjusted models.

### 2.4. Statistical Analyses

The descriptive characteristics of the study sample are shown as means and standard deviations. The differences between MHO and MUO were determined by independent t-tests and chi-squared tests.

To test whether differences existed between MHO and MUO in global gray matter, we performed analysis of covariance (ANCOVA) models with MHO/MUO as a fixed factor and global volume as the dependent variable. Confounders (i.e., sex, peak height velocity, parental education level, and BMI) were selected after examining their influence on the estimates. Thus, the data were presented adjusted for basic confounders, and additionally adjusted for cardiorespiratory fitness. In exploratory analyses (presented in Supplementary Material), we also examined the differences between MHO and MUO in global white matter and total brain volume. All statistical procedures (t-test, chi-square, and ANCOVA) were performed using IBM SPSS Statistical Software (version 20 for Windows, IBM, Armonk, NY, USA) with an alpha level of 0.05.

For regional gray matter volume, we performed general linear model analyses using the SPM12 software (Wellcome Department of Cognitive Neurology, London, United Kingdom). The difference in gray matter volume between MHO and MUO was analyzed using factor models, adjusted for sex, peak height velocity, parental education level, and BMI (Model 1). We additionally adjusted for cardiorespiratory fitness (Model 2). For those brain regions that showed statistical significance, we extracted the eigenvalues of each significant cluster (*k*). The effect size (Cohen’s d and its 95% confidence intervals) was calculated for each extracted cluster. The statistical threshold in the regional gray matter analyses was calculated with AlphaSim in Resting-State fMRI Data Analysis Toolkit toolbox plus v1.2 (RESTplus) [32]. The parameters were defined as follows: cluster connection radius (rmm)  =  5 mm, the smoothness of the data after model estimation and applying a gray matter mask with a volume of 128,190 voxels. The voxel-level alpha significance (threshold, *p* < 0.001 uncorrected), along with the appropriate cluster size for controlling for multiple comparisons in each analysis, was indicated in the results. The resulting cluster extents were further adjusted, as described by Hayasaka et al. [33], to account for the non-isotropic smoothness of structural images. In sensitivity analyses, we further analyzed the differences between metabolically healthy and unhealthy only in the obese sample.

Lastly, to examine the associations between gray matter volume and academic achievement, we performed a linear regression analysis, where the total academic achievement was used as the dependent variable and gray matter volume as an independent variable. Model 1 was adjusted for basic confounders (i.e., sex, age, parental education level, and BMI), and Model 2 was additionally adjusted for cardiorespiratory fitness. 

## 3. Results

The descriptive characteristics of the study sample are shown in Table 1. Briefly, MHO children demonstrated lower peak height velocity, weight, and BMI compared to MUO children (all *p* ≤ 0.031). Furthermore, MHO individuals were fitter than MUO (*p* = 0.001). No significant differences were found for most metabolic risk factors (all *p* ≥ 0.383), except for triglycerides and high-density lipoprotein, which showed a better metabolic profile for MHO compared to MUO individuals (all *p* < 0.001). Lastly, MHO showed higher academic achievement scores than their MUO peers (*p* = 0.028).

**Figure 1:**
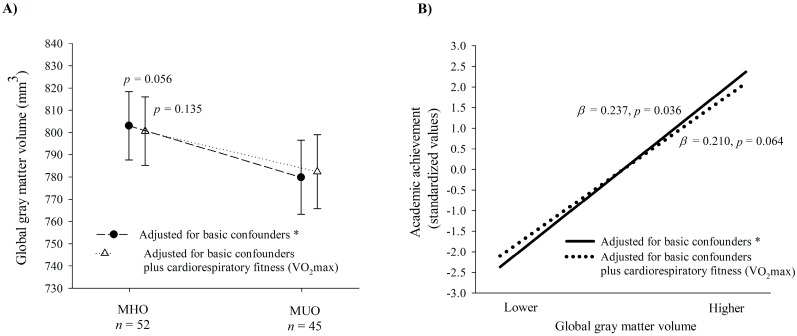
Differences in global gray matter between metabolically healthy and metabolically unhealthy overweight/obesity (Panel **A**) and associations between global gray matter and academic achievement (Panel **B**). *β*: beta standardized coefficients. MHO: Metabolically healthy overweight/obesity. MUO: Metabolically unhealthy overweight/obesity. VO_2_max: maximum oxygen consumption. * Basic confounders were sex, peak height velocity (Panel **A**) or age (Panel **B**), parental education level (none/one/both of them), and body mass index (kg/m^2^).

Figure 1A depicts differences between MHO and MUO in global gray matter volume. After adjusting for basic confounders (i.e., sex, peak height velocity, parental education level, and BMI), the difference was marginally significant (mean difference, MHO minus MUO = 23.24 mm^3^, *p* = 0.056). Finally, when cardiorespiratory fitness was included in the model, this difference in gray matter volume between MHO and MUO was no longer trending (mean difference = 18.19 mm^3^, *p* = 0.135). Sensitivity analyses showed that there were no significant differences between metabolically healthy and unhealthy when only obese children were included (Figure S1A).

**Figure 2:**
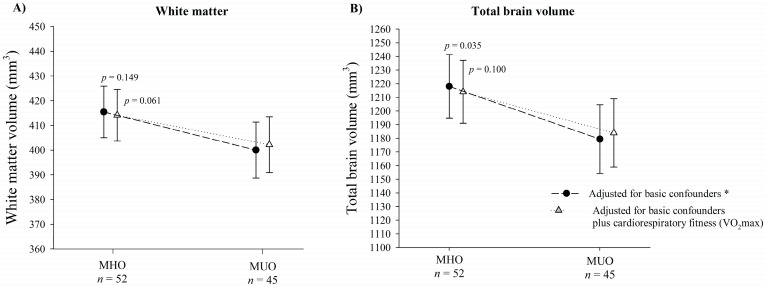
Differences in global white matter (**A**) and total brain volume (**B**) between metabolically healthy and metabolically unhealthy overweight/obesity. MHO: Metabolically healthy overweight/obesity. MUO: Metabolically unhealthy overweight/obesity. * Basic confounders were sex, peak height velocity (years), parental education level (none/one/both of them), and body mass index (kg/m^2^).

Exploratory analyses revealed a similar pattern for global white matter volume (Figure 2A). MHO showed significantly greater total brain volume compared to MUO after adjustment for basic confounders (mean difference = 38.64 mm^3^, *p* = 0.035), yet this difference became non-significant after additional adjustment for cardiorespiratory fitness (mean difference = 30.13 mm^3^, *p* = 0.100) (Figure 2B).

Table 2 shows the brain regions that depicted metabolic differences (i.e., MHO > MUO) in gray matter volume adjusted for basic confounders (Model 1: sex, peak height velocity, parental education level, and BMI) and cardiorespiratory fitness (Model 2). MHO children showed greater gray matter volume in six cortical regions (i.e., bilateral fusiform gyrus, bilateral calcarine, bilateral lingual gyrus, right middle occipital gyrus, right superior temporal gyrus, and left inferior temporal gyrus) after adjusting for basic confounders (all *p* < 0.001; *k* > 60). When cardiorespiratory fitness was added to the model (Model 2), the associations were generally attenuated or disappeared (being that four out of six regions remained significant although having a reduction in the Cohen’s d effect size, d ≥ 0.1) in each cortical region.

**Figure 3:**
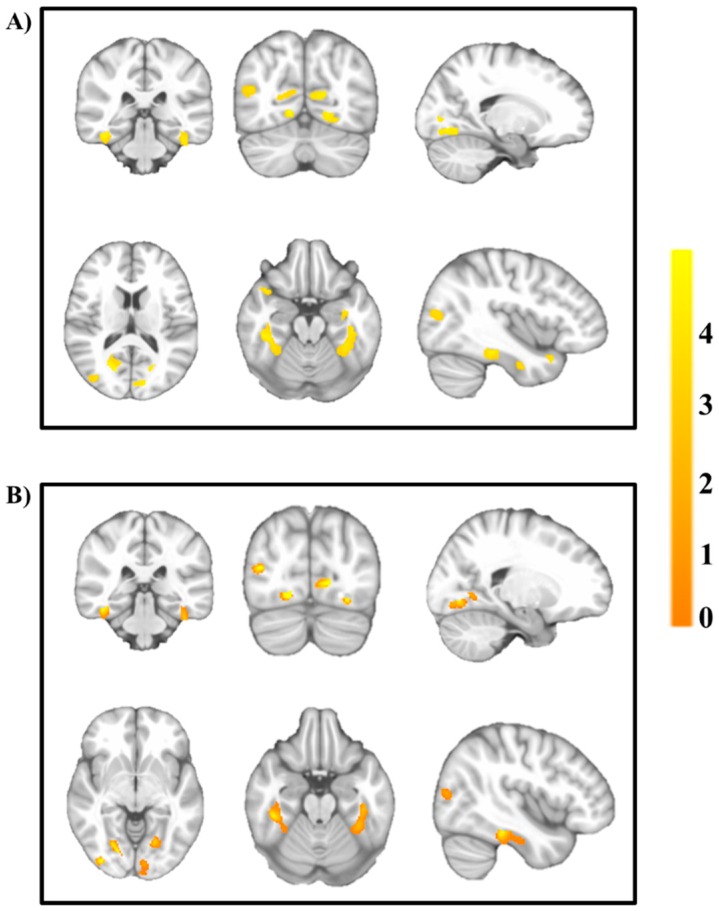
Brain regions showing greater gray matter volume in metabolically healthy overweight/obesity children (*n* = 52) compared to those metabolically unhealthy overweight/obesity (*n* = 45). The analyses were adjusted for sex, peak height velocity (years), parental education level (none/one/both of them), and body mass index (kg/m^2^) (Panel **A**), and additionally for cardiorespiratory fitness (VO_2_max) (Panel **B**). The results were thresholded using AlphaSim at *p* < 0.001 with *k* = 60 (Model 1) and *k* = 54 (Model 2) voxels, and they surpassed Hayasaka correction (see Table 2). The color bar represents the associations (orange color changed to yellow as the association is getting higher, i.e., a greater intensity in the orange color indicated a lower association). The images are displayed in neurological convention; thus, the right hemisphere corresponds to the right side in coronal displays. The sagittal planes show the left hemisphere (top of the figure) and right hemisphere (bottom of the figure).

Figure 3 depicts the results observed for both models (Model 1,2 refer to Panel A,B, respectively), illustrating how the orange color changed to yellow as the association becomes higher (i.e., a greater intensity in the orange color indicated a lower association). MUO did not show greater regional gray matter volume compared to MHO (*p* < 0.05). We performed sensitivity analyses by restricting the sample to only obese children, and the results showed that only two regions were significantly different between metabolically healthy and unhealthy participants (i.e., lingual and fusiform gyrus) after adjusting for basic confounders and for cardiorespiratory fitness (Table S2).

Figure 1B shows the results from a linear regression analysis between gray matter volume and academic achievement. We found positive associations between global gray matter volume and academic achievement (*β* = 0.237, *p* = 0.036) after adjusting for basic confounders (i.e., sex, age, parental education level, and BMI). When cardiorespiratory fitness was added to the model, the relationship between gray matter and academic achievement was borderline non-significant (*β* = 0.210, *p* = 0.064). Sensitivity analyses showed similar results in only obese children (Figure S1B). In regard to regional gray matter volume (Table 3), no significant associations were found for any of the coordinates examined after adjusting for basic confounders (all *β* ≤ 0.188, *p* > 0.05) and additionally by cardiorespiratory fitness (all *β* ≤ 0.104, *p* > 0.275). Exploratory analysis showed that total brain volume was positively associated with academic achievement after adjusting for basic confounders (*β* = 0.248, *p* = 0.036) and that this association disappeared when cardiorespiratory fitness was additionally included as a covariate (*β* = 0.218, *p* = 0.068) (Figure S2).

## 4. Discussion

The main findings of this study were the following: (1) MHO children showed marginally greater global gray matter volume and significantly greater total brain volume compared to MUO children; (2) MHO children had greater gray matter in six cortical brain structures (i.e., bilateral fusiform gyrus, bilateral calcarine, bilateral lingual gyrus, right middle occipital gyrus, right superior temporal gyrus, and left inferior temporal gyrus) compared to MUO children; (3) global gray matter volume (but not regional) and total brain volume were positively associated with academic achievement; and (4) all observed differences were attenuated or disappeared after adjusting for cardiorespiratory fitness, suggesting a role for fitness in the association of the MHO phenotype with brain structure and academic achievement. These results contribute to the existing knowledge by investigating, for the first time, global and regional brain volume differences in MHO and MUO children and the relationship with academic achievement. Furthermore, we shed light on the clearly important role of cardiorespiratory fitness in these associations.

In the last few years, an increased body of evidence has emerged on obesity-related comorbidities and metabolic alterations and their relationship with structural brain abnormalities in children and adolescents [9,34,35,36]. For instance, Perlaki et al. [36] showed that a higher degree of obesity was associated with greater volumes in the amygdala and accumbens, regions involved in the food rewards. Interestingly, in our study, we observed that, in those who presented a MHO phenotype, global gray matter volume was marginally greater compared to those with a metabolically unhealthy profile. Moreover, we observed a similar trend for global white matter and observed significantly greater total brain volume in MHO compared to MUO, after adjusting for relevant basic confounders, such as sex, age, parental education level, and BMI. Of note, MUO had higher BMI than MHO in this study, which could explain a potential relationship of higher body size with larger brain volumes. However, the difference between groups was small (around 2 km/m^2^) and BMI did not show associations with gray matter volume in this sample in a previous study [9]. Thus, its confounding effect in this study associations does not appear to be relevant. Nevertheless, we showed that MHO exhibited greater volumes than MUO after removing the confounding effect of BMI. Specifically, MHO children had greater gray matter volume in six cortical brain structures (i.e., bilateral fusiform gyrus, bilateral calcarine, bilateral lingual gyrus, right middle occipital gyrus, right superior temporal gyrus, and left inferior temporal gyrus) compared to MUO children. However, all these differences were attenuated or disappeared after accounting for cardiorespiratory fitness, suggesting that cardiorespiratory fitness might play a role in the relationship between MHO and the brain. In regard to regional brain analyses, our results indicated that MHO was associated with greater gray matter in six cortical brain regions (i.e., bilateral fusiform gyrus, bilateral calcarine, bilateral lingual gyrus, right middle occipital gyrus, right superior temporal gyrus, and left inferior temporal gyrus) compared to MUO. It is interesting to note that the differences found between MHO and MUO were only in the temporal lobe. The frontal lobe has previously been found to be sensitive to weight status in adults [8,37]. Different age groups and homogeneous weight status (all children in our sample had overweight or obesity status) could explain the lack of differences in the frontal lobe. As occurred with global gray matter volume, after adjusting for cardiorespiratory fitness, there was an attenuation in the number of regions meeting statistical threshold (from six to four cortical brain regions).

The lack of prior studies examining the differences between metabolic phenotypes and global and regional brain volume limits our ability to compare these results with other studies. However, several pathways and potential mechanisms could interfere and explain our novel findings. Differences in visceral fat accumulation, birth weight, adipose cell size, and gene expression-encoding marker of adipose [38] health differentiation may favor the development of a healthy metabolic phenotype. Consequently, our results might be explained by lower levels of inflammation and insulin resistance [18,39], and also lower levels of growth factors, such as brain derived neurotrophic factor (BDNF), vascular endothelial growth factor (VEGF), and insulin-like growth factor (IGF), which contribute to gray matter development [40] and are also related to cardiorespiratory fitness. Our findings should be considered when promoting strategies among children to prevent future brain health impairment. However, such speculation should be taken with caution since this study is the first to analyze both global and regional gray matter volume based on metabolic phenotypes (i.e., MHO and MUO).

Another important aspect of the present study was the analysis examining the relationship between gray matter volume and academic achievement among overweight and obese youth, observing both a positive association (global gray matter) and no association (regional gray matter). In a previous study, also from the ActiveBrains project, we observed that, in regional analysis, gray matter volume in the hippocampus (previously associated with cardiorespiratory fitness) was also positively related to total academic achievement in overweight/obese children [22]. In the present study, we add to the previous literature [22] by showing a positive association between global gray matter, total brain volume, and academic achievement, in addition to the regional associations previously shown (i.e., bilateral fusiform gyrus, left calcarine, bilateral lingual gyrus, and right middle occipital gyrus). However, the reason why we did not find any significant association in the current study between regional gray matter volume and academic achievement could be due to the different functionality of the regions examined being not strongly related with improving executive function, memory, or intelligence, which in turn could improve academic achievement. Nevertheless, more studies examining the association between global and regional gray matter volume are needed in order to corroborate or contrast our findings. The role of cardiorespiratory fitness in the context of metabolic phenotypes (i.e., MHO and MUO) has been previously examined in relation to several health outcomes [2,16,20,21]. Our group recently showed that the higher risk in cardiovascular prognosis observed in MHO adults compared to metabolically healthy normal-weight is explained by cardiorespiratory fitness [17,21]. In the present study, our results showed no significant differences between metabolic phenotypes in global gray matter volume. Furthermore, differences in only four out of six brain regions remained statistically significant after controlling for cardiorespiratory fitness. It is interesting to highlight that those regions that no longer remained significant were strongly associated in our previous study with cardiorespiratory fitness in overweight/obese children [22]. However, our previous study also showed associations in the fusiform gyrus and calcarine with cardiorespiratory fitness [22], while our results showed that these areas remained significant although with a reduction of 48.6 to 73% of the cluster size after controlling for cardiorespiratory fitness. Of note, the non-significant differences found in global gray matter were not exclusively related to the inclusion of cardiorespiratory fitness in the model, but also due to the inclusion of basic confounders, which resulted in only marginally significant effects. Yet, in an exploratory analysis, when global structure volumes were combined together (i.e., total brain volume), our results also showed the role of fitness between metabolic phenotypes. Interestingly, concerning regional brain volume, the four brain regions (i.e., bilateral fusiform gyrus, left calcarine, bilateral lingual gyrus, and right middle occipital gyrus) showed a reduced cluster size and effect size in MUO in comparison to MHO after controlling for cardiorespiratory fitness. In accordance with these findings, the association between gray matter and total brain volume and academic achievement was also attenuated or disappeared when we included cardiorespiratory fitness in the model. 

Previous studies have tested the ‘fat but fit’ paradox, showing that higher levels of fitness attenuate the negative consequences of excess body mass on cardiovascular disease risk [2,41]. Nevertheless, whether fitness also attenuates the adverse consequences of obesity on other outcomes, such as brain and brain-related outcomes, remains poorly understood. Relatedly, Opel et al. [42] found a significant mediation effect of fitness on cognitive performance through white matter, which further points to a crucial role of brain structural alternation in the association between fitness and cognition in healthy young adults. Our findings add to this body of knowledge by supporting that there are differences in the brain between MHO and MUO children and that these differences are at least partially explained by cardiorespiratory fitness. Our data also suggest that the association of global gray matter and total brain volume with academic achievement was attenuated or disappeared after accounting for cardiorespiratory fitness. 

The main limitations of this study are the following: (1) the cross-sectional design does not allow for causal inference; (2) the limited sample size, especially in children with obesity (and therefore used only for sensitivity analyses, see Supplementary Material), reduces the power of the analyses; and (3) the lack of a normal-weight group precludes us from comparing overweight/obese with normal-weight children. The strengths of the present study are the novelty of the investigation, the use of magnetic resonance imaging to assess brain structure in nearly 100 children, and the use of a thorough (roughly 2 h testing per child) and standardized academic achievement test.

## 5. Conclusions

Our results support the argument that MHO children have greater gray matter and total brain volume than their MUO peers, which was in turn related to better academic achievement in children aged 8–11-years-old. These findings support the notion that metabolic abnormalities might negatively influence brain development in a sensitive period of growth. Our data also indicate that cardiorespiratory fitness might play an important role in the differences observed in the brain between both metabolic phenotypes. The fact that metabolic dysregulation appears to influence brain structure at this age level is highly relevant and might further underline the need for preventive measures. Therefore, the present study provides additional novel evidence on the importance of having a healthy metabolic profile and high cardiorespiratory fitness level in overweight/obese children. Future studies examining the relationships between metabolic phenotypes and brain health in youth are needed to corroborate and extend our findings.

## Figures and Tables

**Table 1:** Descriptive characteristics of the study sample (*n* = 97).

	All (*n* = 97)	MHO (*n* = 52)	MUO (*n* = 45)	*p*
	Mean ± SD	Mean ± SD	Mean ± SD	
Descriptive characteristics:				
Age (years)	10.0 ± 1.2	9.9 ± 1.1	10.1 ± 1.2	0.473
Peak height velocity (years)	−2.0 ± 1.0	−2.2 ± 1.0	−1.7 ± 0.9	**0.031**
Weight (kg)	56.0 ± 11.1	53.4 ± 11.2	59.0 ± 10.1	**0.011**
Height (cm)	144.1 ± 8.3	143.3 ± 8.4	144.9 ± 8.2	0.325
Body mass index (kg/m^2^)	26.8 ± 3.7	25.7 ± 3.5	28.0 ± 3.5	**0.003**
Weight status (*n*, (%)) *:				**0.016**
Overweight	24 (24.7)	19, (36.5)	5, (11.1)	
Obesity type I	42 (43.3)	21, (40.4)	21, (46.7)	
Obesity type II	19 (19.6)	6, (11.5)	13, (28.9)	
Obesity type III	12 (12.4)	6, (11.5)	6, (13.3)	
Parental education (*n* (%)):				0.248
None with university studies	65 (67.0)	31 (59.6)	34 (75.6)	
Only one with university studies	17 (17.5)	11 (21.2)	6 (13.3)	
Both of them with university studies	15 (15.5)	10 (19.2)	5 (11.1)	
Cardiorespiratory fitness:				
Cardiorespiratory fitness (VO_2_max) ^†^	40.8 ± 2.7	41.6 ± 2.6	39.8 ± 2.6	**0.001**
Metabolic risk factors:				
Triglycerides (mg/dL)	98.6 ± 57.7	73.3 ± 23.7	128.6 ± 72.8	**<0.001**
Glucose (mg/dL)	86.3 ± 6.6	86.4 ± 5.8	86.4 ± 7.5	0.987
High-Density Lipoprotein (mg/dL)	50.3 ± 11.2	56.7 ± 9.7	42.6 ± 9.6	**<0.001**
Systolic blood pressure (mmHg)	99.6 ± 12.9	98.9 ± 10.7	100.9 ± 15.2	0.458
Diastolic blood pressure (mmHg)	56.0 ± 12.3	55.2 ± 10.4	57.4 ± 14.1	0.383
Academic achievement:				
Total achievement	108.8 ± 12.4	111.38 ± 12.9	105.9 ± 11.2	**0.028**

MHO: Metabolically healthy overweight/obesity. MUO: Metabolically unhealthy overweight/obesity. SD: Standard deviation. Data are presented as mean and standard deviations unless otherwise indicated. Statistically significant values are shown in bold. * Classified according to Cole et al. [25] and Bervoets et al. [24]. ^†^ Measured by the 20-m shuttle run test, estimated following the Leger et al. [30] equation.

**Table 2:** Brain regions showing gray matter volume increases in metabolically healthy overweight/obesity compared to metabolically unhealthy overweight/obesity (*n* = 97).

MHO (*n* = 52) > MUO (*n* = 45)								
Brain Regions	x	y	z	t	Cluster Size	Hemisphere	Effect Size	
							Cohen’s d	95% CI
**Model 1:**							
Fusiform gyrus	44	−33	−20	4.33	2008	Right	0.82	0.23, 1.05
	−41	−30	−27	4.35	1581	Left	0.57	0.16, 0.98
Calcarine	−12	−83	2	4.33	948	Left	0.72	0.31, 1.13
	18	−66	14	3.69	662	Right	0.61	0.20, 1.01
Lingual gyrus	−20	−68	−5	4.42	893	Left	0.67	0.26, 1.08
	20	−77	−6	4.85	386	Right	0.80	0.38, 1.21
Middle occipital gyrus	41	−80	14	3.72	120	Right	0.57	0.17, 0.98
Superior temporal gyrus	36	20	−33	3.99	93	Right	0.64	0.23, 1.05
Inferior temporal gyrus	−38	−6	−35	3.74	76	Left	0.53	0.12, 0.93
**Model 2:**								
Fusiform gyrus	−41	−30	−27	4.08	700	Left	0.39	0.06, 0.79
	44	−33	−20	4.64	427	Right	0.68	0.27, 1.09
Calcarine	−12	−83	2	4.19	487	Left	0.58	0.17, 0.98
Lingual gyrus	−20	−68	−5	4.15	388	Left	0.50	0.09, 0.90
	20	−75	−6	4.62	256	Right	0.62	0.20, 1.02
Middle occipital gyrus	41	−78	12	3.67	94	Right	0.47	0.06, 0.87
Superior temporal gyrus	ns	ns	ns	ns	ns	ns	ns	ns
Inferior temporal gyrus	ns	ns	ns	ns	ns	ns	ns	ns

MHO: Metabolically healthy overweight/obesity. MUO: Metabolically unhealthy overweight/obesity; ns: No significant difference. 95% CI: 95% of confidence interval. Model 1 was adjusted by sex, peak height velocity (years), parental education level (none/one/ both of them), and body mass index (kg/m^2^). Model 2 was adjusted for model 1 plus cardiorespiratory fitness (VO_2_max). All contrasts were thresholded using AlphaSim at *p* < 0.001 with *k* = 60 voxels (Model 1) and *k =* 54 voxels (Model 2) and surpassed Hayasaka correction. Anatomical coordinates (x, y, z) are given in Montreal Neurological Institute (MNI) Atlas space.

**Table 3:** Associations between regional gray matter volume and academic achievement in overweight and obese children.

				Model 1		Model 2	
	Coordinates (x, y, z)			*β*	*p*	*β*	*p*
Regional gray matter:							
Fusiform gyrus	44	−33	−20	0.171	0.065	−0.080	0.395
	−41	−30	−27	0.034	0.713	0.062	0.514
Calcarine	−12	−83	2	0.006	0.959	0.104	0.275
	18	−66	14	−0.012	0.901	-	-
Lingual gyrus	−20	−68	−5	−0.188	0.052	0.076	0.433
	20	−77	−6	0.042	0.672	0.028	0.772
Middle occipital gyrus	41	−80	14	0.030	0.751	−0.071	0.473
Superior temporal gyrus	36	20	−33	0.094	0.350	-	-
Inferior temporal gyrus	−38	−6	−35	0.139	0.167	-	-

*β*: beta standardized coefficients. Model 1 was adjusted by sex, age, parental education level (none/one/ both of them), and body mass index (kg/m^2^). Model 2 was adjusted for Model 1 plus cardiorespiratory fitness (VO_2_max).

## Supplementary Materials

The following are available online at https://www.mdpi.com/2077-0383/9/4/1059/s1, Figure S1: Differences in global gray matter between metabolically healthy and metabolically unhealthy obese (Panel A) and associations between global gray matter and academic achievement (Panel B) in only obese children. Figure S2: Associations between total brain volume and academic achievement in overweight and obese children. Table S1: Jolliffe and Janssen [10] criteria for classification of metabolically healthy and unhealthy phenotypes. Table S2: Brain regions showing gray matter volume increases in metabolically healthy (*n* = 33) compared to metabolically unhealthy (*n* = 40) obese children.
