# Supplementary material for: Differences in Brain Volume between Metabolically Healthy and Unhealthy Overweight and Obese Children: The Role of Fitness
Source: J Clin Med. 2020 Apr 8;9(4):1059. doi: 10.3390/jcm9041059 (PMC7231256; doi:10.3390/jcm9041059)
Supplement: Supplementary file 1 [file jcm-09-01059-s001.pdf]

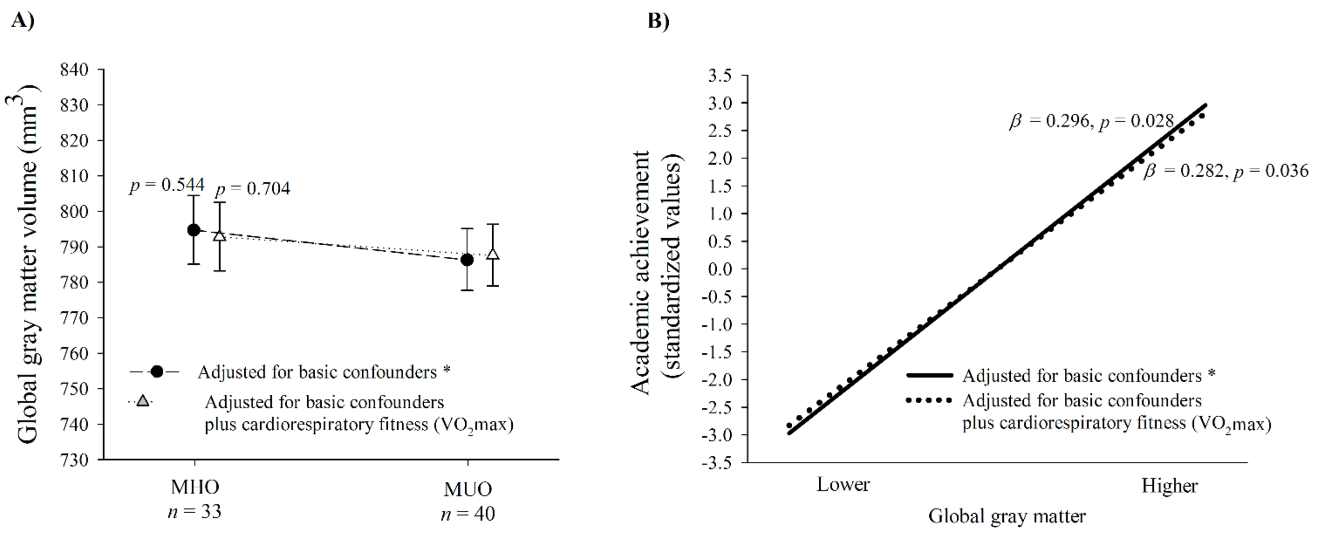

Figure S1. Differences in global gray matter between metabolically healthy and metabolically unhealthy obese (Panel **A**) and associations between global gray matter and academic achievement (Panel **B**) in only obese children. β: beta standardized coefficients. MHO: Metabolically healthy overweight/obesity. MUO: Metabolically unhealthy overweight/obesity. VO_2max: maximum oxygen consumption. * Basic confounders were sex, peak height velocity (Panel A) or age (Panel B), parental education level (none/one/both of them), and body mass index (kg/m^2).

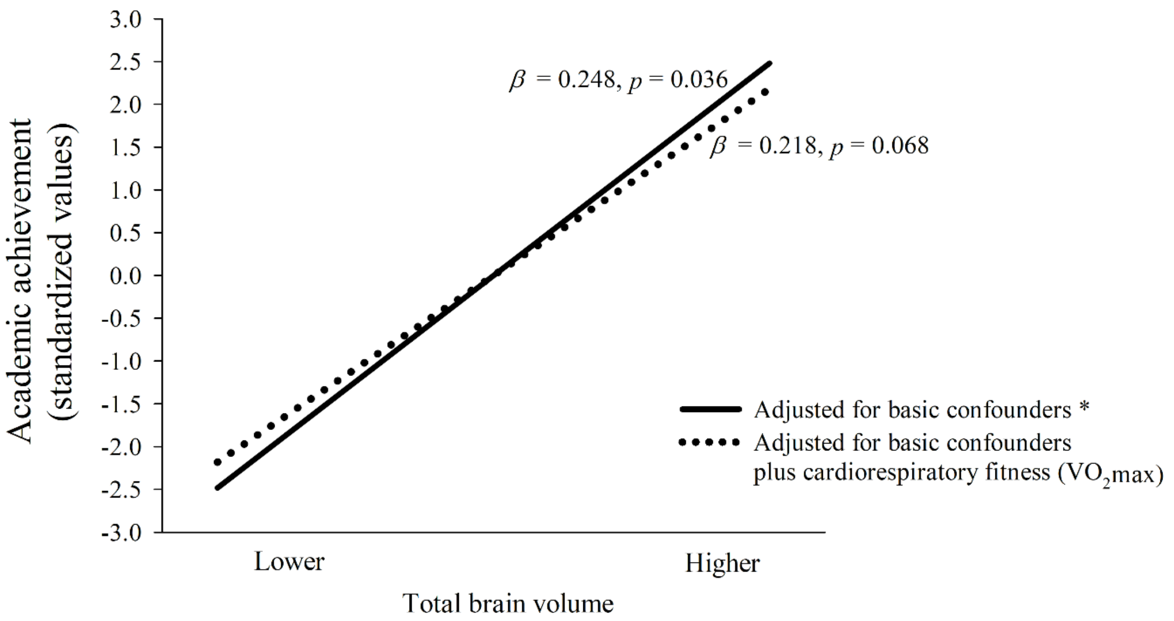

Figure S2. Associations between total brain volume and academic achievement in overweight and obese children. β = beta standardized coefficients. * Basic confounders were sex, age, parental education level (none/one/both of them), and body mass index (kg/m^2).

Table S1. Jolliffe and Janssen [10] criteria for classification of metabolically healthy and unhealthy phenotypes.

Years	Waist Circumference (cm)	Triglycerides (mg/dL)	Glucose (mg/dL)	HDL Cholesterol (mg/dL)	Systolic Blood Pressure (mmHg)	Diastolic Blood Pressure (mmHg)
**Boys:**						
12	≥85.1	≥127.40	≥100.97	≥43.70	≥121	≥76
**Girls:**						
12	≥72.5	≥141.60	≥100.97	≥48.34	≥121	≥80

HDL: High-density lipoprotein.

Table S2. Brain regions showing gray matter volume increases in metabolically healthy (n = 33) compared to metabolically unhealthy (n = 40) obese children.

**Metabolically healthy (n = 33) > Metabolically unhealthy (n = 40)**								
**Brain Regions**	**x**	**y**	**z**	**t**	**Cluster Size**	**Hemisphere**	**Effect Size**	
							Cohen's d	95% CI
**Model 1:**								
Lingual gyrus	23	−72	−6	4.04	111	Right	0.89	0.40,1.36
	−18	−69	−3	3.62	35	Left	0.77	0.28,1.24
Fusiform gyrus	44	−33	−20	3.76	53	Right	0.88	0.39,1.35
**Model 2:**								
Lingual gyrus	23	−72	−6	3.68	70	Right	0.80	0.31,1.27
Fusiform gyrus	44	−33	−20	3.49	37	Right	0.80	0.31,1.27

95% CI: 95% of confidence interval. Model 1 was adjusted by sex, peak height velocity (years), parental education level (none/one/both of them), and body mass index (kg/m^2). Model 2 was adjusted for model 1 plus cardiorespiratory fitness (VO_2max). All contrasts were thresholded using AlphaSim at $p < 0.001$ with $k = 33$ voxels (Model 1) and $k = 25$ voxels (Model 2) and surpassed Hayasaka correction. Anatomical coordinates (x, y, z) are given in Montreal Neurological Institute (MNI) Atlas space.
